# Exploring preconception health in adolescents and young adults: Identifying risk factors and interventions to prevent adverse maternal, perinatal, and child health outcomes–A scoping review

**DOI:** 10.1371/journal.pone.0300177

**Published:** 2024-04-17

**Authors:** Zahra Ali Padhani, Komal Abdul Rahim, Gizachew A. Tessema, Jodie C. Avery, Negin Mirzaei Damabi, Patience Castleton, Rehana A. Salam, Salima Meherali, Zohra S. Lassi

**Affiliations:** 1 School of Public Health, Faculty of Health and Medical Sciences, University of Adelaide, Adelaide, SA, Australia; 2 Robinson Research Institute, Faculty of Health and Medical Sciences, University of Adelaide, Adelaide, SA, Australia; 3 Centre of Excellence in Trauma and Emergencies (CETE), Aga Khan University Hospital, Karachi, Pakistan; 4 Dean’s Office, Medical College, Aga Khan University Hospital, Karachi, Pakistan; 5 Centre of Research Excellence, Melanoma Institute Australia, University of Sydney, Sydney, Australia; 6 College of Health Sciences, Faculty of Nursing, University of Alberta, Edmonton Clinic Health Academy, Edmonton, AB, Canada; University of the Witwatersrand, SOUTH AFRICA

## Abstract

**Background:**

Preconception health provides an opportunity to examine a woman’s health status and address modifiable risk factors that can impact both a woman’s and her child’s health once pregnant. In this review, we aimed to investigate the preconception risk factors and interventions of early pregnancy and its impact on adverse maternal, perinatal and child health outcomes.

**Methods:**

We conducted a scoping review following the PRISMA-ScR guidelines to include relevant literature identified from electronic databases. We included reviews that studied preconception risk factors and interventions among adolescents and young adults, and their impact on maternal, perinatal, and child health outcomes. All identified studies were screened for eligibility, followed by data extraction, and descriptive and thematic analysis.

**Findings:**

We identified a total of 10 reviews. The findings suggest an increase in odds of maternal anaemia and maternal deaths among young mothers (up to 17 years) and low birth weight (LBW), preterm birth, stillbirths, and neonatal and perinatal mortality among babies born to mothers up to 17 years compared to those aged 19–25 years in high-income countries. It also suggested an increase in the odds of congenital anomalies among children born to mothers aged 20–24 years. Furthermore, cancer treatment during childhood or young adulthood was associated with an increased risk of preterm birth, LBW, and stillbirths. Interventions such as youth-friendly family planning services showed a significant decrease in abortion rates. Micronutrient supplementation contributed to reducing anaemia among adolescent mothers; however, human papillomavirus (HPV) and herpes simplex virus (HSV) vaccination had little to no impact on stillbirths, ectopic pregnancies, and congenital anomalies. However, one review reported an increased risk of miscarriages among young adults associated with these vaccinations.

**Conclusion:**

The scoping review identified a scarcity of evidence on preconception risk factors and interventions among adolescents and young adults. This underscores the crucial need for additional research on the subject.

## Introduction

Preconception health refers to the health of both men and women during their reproductive years [1, 2]. According to the Developmental Origins of Health and Disease (DOHaD) theory, harmful early-life exposures can increase the risk of diseases in later life [3]. The DOHaD theory demonstrates a connection between the in-utero environment and the risk of developing numerous non-communicable diseases and behavioural disorders in the growing child [3]. Preconception care is vital in providing awareness to ameliorate these risk factors and promoting healthy behaviours to improve health outcomes [2].

Preconception care refers to care before pregnancy to improve the health of both the mother and her child [4]. There is growing evidence referring to the preconception care for women of reproductive age (WRA); it is especially crucial during adolescence and young adulthood [5–11]. These formative periods present growth and disease prevention opportunities across the life course [12]. Lifestyle behaviours, physical changes, and social transitions during adolescence significantly influence the health of individuals and the health of their families and the community [12]. The lifestyle behaviours (e.g., substance misuse, risky sexual activities, unsafe sex, poor nutritious diet, sedentary lifestyle) during adolescence and the onset of puberty can have far-reaching consequences [12, 13]. These consequences include unintended pregnancies, unsafe abortions, malnutrition, sexually transmitted infections, and mental health issues, leading to poor pregnancy and birth outcomes [2, 12].

Research has shown strong links between preconception risk factors with pregnancy and perinatal complications amongst adolescent populations. Substance use, sexual abuse, child marriages, poor socio-economic status, and unsafe sex are major contributors to unintended and unplanned pregnancies. In 2021, an estimated 14% of adolescent women globally, gave birth before the age of 18 [14]. Evidence suggests that adolescent mothers (15–19 years) are 1.4 times more likely to experience pregnancy complications, including pre-eclampsia and antepartum haemorrhage (APH), compared to mothers aged between 20 and 35 years [15]. Moreover, existing evidence also shows a significant increase in odds of maternal anaemia, obstructed labour, stillbirths, congenital anomalies, small for gestational age (SGA), infant mortality, asthma among children aged ≥6 years, learning disability, attention-deficit/hyperactivity disorder and cancer among children born to adolescent mothers [16–22]. While the evidence on pregnancy during early adolescence and later adolescence is yet to be investigated. Other major preconception risk factors among adolescents are mental health, poor nutrition, violence, and non-communicable diseases, which have not been extensively studied [7, 23, 24].

Preconception care has gained immense importance to ensure optimum health for women entering pregnancy as it is evident that preconception risk factors, if not catered at an earlier stage, can have a detrimental impact on maternal and child health [25, 26]. Preconception care involves several health interventions such as risk assessment, health promotion, and behavioural and psychosocial interventions [27, 28]. These interventions include nutrition supplementation and physical exercise, screening and management of chronic diseases, control of tobacco and alcohol use, micronutrient supplementation, immunisation, prevention of sexually transmitted diseases, and contraception education and distribution [5–7, 26, 28–30]. These interventions have shown to reduce adverse maternal, perinatal, and child health outcomes among women of reproductive age but the impact is not yet clear among adolescents [5, 6].

Since the release of the Millenium Development Goals in the year 2000 and the World Health Organisation (WHO) guidelines on preconception health in year 2012, there has been a slight shift of focus from improving maternal health to improving preconception of women of reproductive age [5, 26]. Multiple reviews exist that broadly look at the preconception of women of reproductive age [6, 7, 25, 26, 28, 30], therefore, in this review we aimed to collate existing evidence on preconception health risk factor and interventions among adolescents and young adults to prevent adverse maternal, perinatal, and child health outcomes.

## Methods

This scoping review followed the Preferred Reporting Items for Systematic Reviews and Meta-Analyses extension for Scoping Reviews (PRISMA-ScR) (**S1** Checklist) [31]. The review protocol has been registered on The Open Science Framework (doi: https://doi.org/10.17605/OSF.IO/R2U39).

### Selection criteria

We included systematic reviews, umbrella reviews or overview of reviews, and scoping reviews, which reported the impact of preconception health risk factors and interventions (**Table 1**) on adverse maternal, perinatal, and child health outcomes. We excluded interventional reviews spanning both preconception and gestational periods and reviews that studied the risk factors during the gestational period. Our focus was specifically on studies targeting adolescents and young adults aged 10 to <25 years of age. Studies reporting on WRA aged 15–49 years were excluded; however, studies that reported disaggregated data on adolescents and young adults were included. We included reviews published in English since the year 2010, aligning with the initiation of momentum to improve preconception health started by the WHO to address poor maternal and perinatal outcomes through stakeholder meetings across different regions worldwide [32, 33].

**Table 1:** Preconception risk factor and intervention considered for inclusion.

Risk factors	Interventions
Young maternal age Unsafe sex Communicable and non-communicable diseases/chronic diseases Malnutrition (underweight, overweight, and obesity) Micronutrient deficiencies Anaemia Poor oral health Wounds and injuries Smoking, tobacco, alcohol, and substance use Medication use Infections Intimate partner violence Sexual abuse and domestic violence Mental health issues Chemical exposure and environment Sexually transmitted infections Female genital mutilation	General preconception care Family planning services (including education, birth intervals, and contraceptive distribution) Sex education Vaccination/immunisation Nutrition education and supplementation Micronutrient supplementation Lifestyle modification Social protection programs Health promotion Social support and women empowerment Genetic monitoring and testing Dental care and hygiene practices Smoking prevention and cessation programs Rehabilitation from substance abuse Screening for alcohol and substance use Vocational training for youth development and empowerment Drug therapy Counselling Screening and disease management Telemedicine Prevention of environmental risks Prevention of infections Psychological interventions Mental health programs Prevention and treatment of sexually transmitted infections

We included reviews if they reported on maternal (such as pregnancy complications, mode of delivery, weight gain, anaemia, the incidence of sexually transmitted infections (STI)/ human immunodeficiency virus (HIV) or STI-HIV acquisition during pregnancy, and maternal morbidity and mortality), perinatal (such as miscarriage/spontaneous abortions, stillbirths, perinatal and neonatal mortality, preterm birth, small/large for gestation age (SGA/LGA), intrauterine growth retardation (IUGR), mean birthweight, low birthweight (LBW), macrosomia, and congenital anomalies or birth defects), and infant and child health outcomes (such as infant and under-five child mortality, morbidity, mother-to-child STI transmission, poor growth outcomes, physical and/or mental developmental outcomes).

### Search and study selection

A search strategy was formulated using keywords and medical subject headings (MeSH) terms relevant to adolescents and young adults, preconception risk factors and interventions (search strategy in **S1** Text). Searches were conducted on Medline, Embase, CINAHL, PsycINFO, and CENTRAL databases. We also searched the bibliographies of the included studies to identify any potentially overlooked studies during the initial search. The last date of the search was November 2022. We extended our search to Google Scholar on 30^th^ October 2023 for any recently published reviews.

All studies identified in the electronic search were imported into Covidence and subjected to de-duplication. Two reviewers independently screened for titles/abstracts and full texts. Discrepancies were resolved through discussion until a consensus was reached, and a third reviewer was consulted if consensus couldn’t be achieved.

### Data extraction and result synthesis

Following full-text screening, data extraction of included studies was carried out independently by two reviewers on an Excel sheet, and discrepancies were resolved through discussion or consultation with a third reviewer. Data was extracted using a priori data abstraction checklist, including study characteristics (author name, publication year, journal, aims, setting, design, number of included studies), population characteristics (number, age, gender, ethnicity), preconception risk factors and interventions, intervention delivery platform, outcomes, and their effect estimates (when applicable), and study limitations. Thematic and descriptive analyses were conducted for all included studies based on the risk factors studied and interventions provided.

## Results

**Fig 1:**
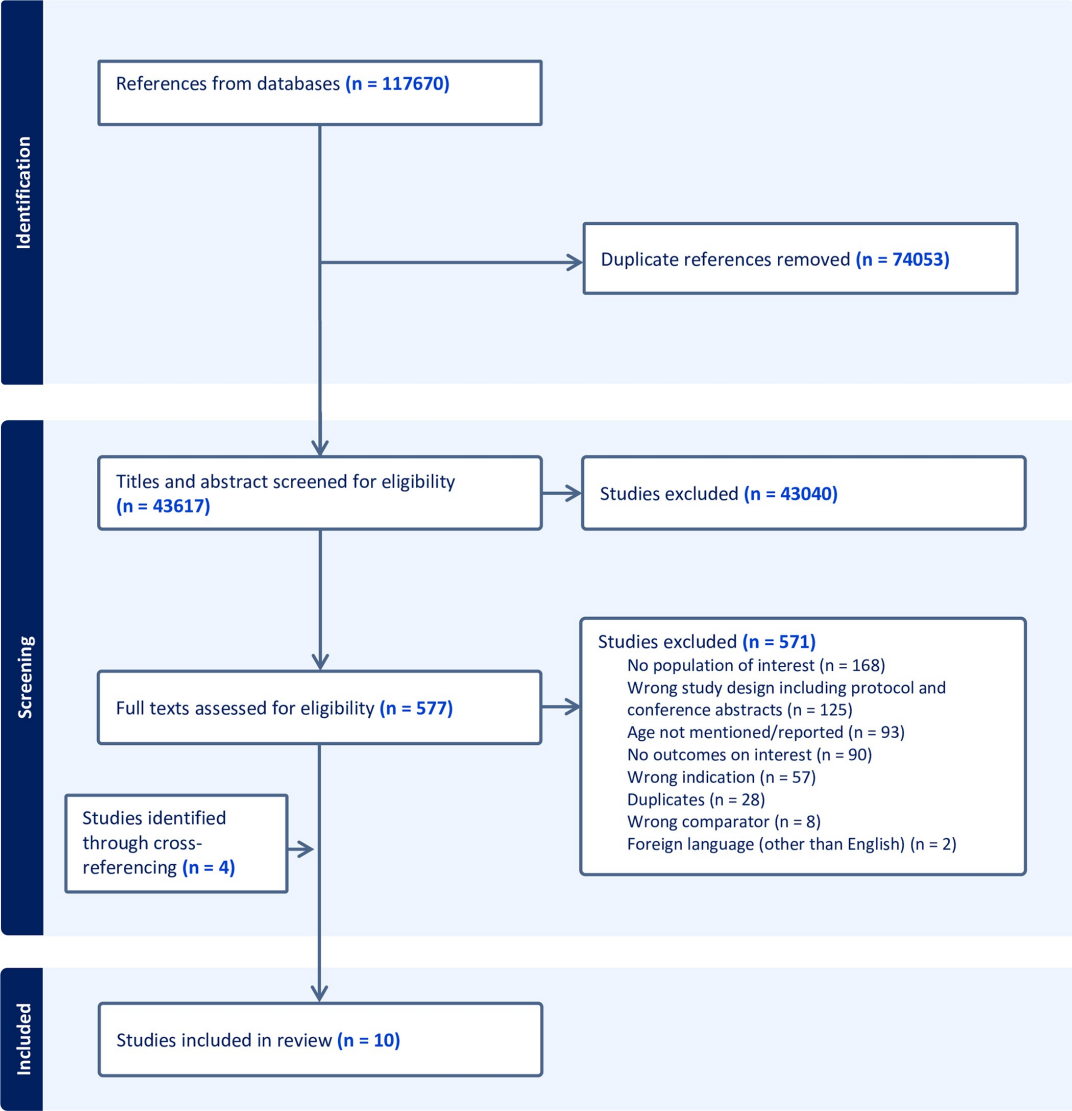
PRISMA flow diagram.

The review initially identified 117670 documents through a database search. After removing duplicates, 43617 studies underwent title and abstract screening, followed by the full-text screening of 577 studies. Four studies were identified through cross-referencing, and ultimately, 10 reviews met the eligibility criteria and were included for data extraction and analysis [34–43]. Most reviews were excluded during the full-text review due to the concentrated focus on the preconception health of women of reproductive age, lacking separate data for adolescents and young adults (**Fig 1**).

All the included studies were systematic reviews (**Table 2**). Four included reviews focused on adolescents and young adults, and the remaining six focused on WRA, providing separate data for adolescents and young adults. Four reviews focused on both high-income countries (HICs) and low- and middle-income countries (LMICs); however, one review each focused on upper-middle-income countries (UMIC), and LMICs only, while one failed to report on the setting. Additionally, the setting of three reviews remains unclear due to inclusion of multi-country trials in which the country is not specified clearly.

**Table 2:** Characteristics of included studies.

Study	Study design	Setting	Participants	Risk factors	Intervention	Comparison	Maternal Outcomes	Perinatal and Pregnancy Outcomes	Infant and Child outcomes
**Preconception health risk factors**									
Young maternal age									
Cardwell 2010*[36]	Systematic review (n = 32 studies; 30 meta-analysed)	HIC and LMIC	Women <20 years to ≥35 years	Maternal age < 20 years vs 20–25 years	NR	NR	NR	NR	Childhood Type 1 Diabetes
Gibbs 2012*[39]	Systematic review (n = 43 studies)	Multi-country (mostly HIC and MIC)	Mothers with chronological age ≤16 years at conception or delivery	Maternal age ≤ 16 years	NR	NR	Anaemia, weight gain during pregnancy	Pre-eclampsia, maternal lacerations, premature rupture of membranes, LBW, PTB, stillbirth, perinatal mortality/early neonatal death	Neonatal mortality (late)
Gronvik 2018*[40]	Systematic review (n = 18 studies; 11 meta-analysed)	Sub Saharan Africa	Adolescents aged 17 years or younger, and with a comparison group of adult women aged between 20 and 35 years	Young maternal age	NR	NR	Maternal mortality	Pre-eclampsia, LBW, preterm birth, stillbirths, SGA	NR
Liu 2016*[41]	Systematic review (n = 11 studies included and meta-analysed)	LMIC and HIC	Women with maternal age of <20 to ≥ 35 years at conception rather than delivery	Maternal age <20 to 24 years.	NR	NR	NR	NR	Club foot
Non-Communicable and Chronic Diseases									
Gerstl 2018[38]	Systematic review (n = 17 studies; 10 studies pooled for analysis)	Multiple countries	Females diagnosed with childhood (0–14 years) or adolescent and young adult (AYA) (15–25 years) cancer between the ages of 0 and 25 years	Cancer history in childhood and adolescent and young adult (AYA)	NR	NR	NR	Stillbirth, LBW, PTB	NR
**Preconception health interventions**									
Nutrition Supplementation and Physical Activity									
Abe, 2016*[34]	Systematic review (n = 2 studies- of which only one study was focused on adolescents)	HIC and LMIC	Non-pregnant adolescent mothers who exclusively fed breast milk or practiced mixed feeding (breast milk and formula)	NA	Studies of multiple-micronutrient supplements of three or more micronutrients	Placebo, no supplementation, or supplementation with two or fewer micronutrients, irrespective of dosage of micronutrient	Maternal anaemia	NR	NR
Family planning									
Brittain 2015[35]	Systematic review (n = 19 studies)	Countries outside the U.S., Canada, Europe, Australia, or New Zealand	Young people (10–24 years)	NA	Youth-friendly family services	Control or standard of care	NR	Abortion rate	NR
Vaccination									
Coelho 2015[37]	Systematic review (n = 14 trials)	Multicentre studies, including one from South Korea	NR	Subjects under 18 years of age	Recombinant human papillomavirus types 6, 11, 16 and 18 vaccine	Control	NR	Dysfunctional uterine bleeding, prematurity, congenital anomaly, late fetal death, miscarriage	NR
Wacholder 2010[43]	Systematic review (n = 2 trials)	Costa Rica	Women aged 15–25 (n = 26130 women; 3599 pregnancies)	NA	Three doses of bivalent HPV 16/18 VLP vaccine with AS04 adjuvant (n = 13,075) for 6 months	Hepatitis A vaccine (HAV) (as control; n = 13,055)	NR	Miscarriage and stillbirths	NR
Tavares 2013*[42]	Systematic review (five trials got meta-analysed out of 19 eligible trials)	NR	Women of reproductive age (mean age 23.6 ± 10.3 years in the intervention arm and 22.0 ± 9.3 years in the control arm); (n = 19,727 vaccinated women; 660 pregnant)	NA	AS04-adjuvanted herpes simplex virus (HSV) glycoprotein D candidate prophylactic vaccine against genital herpes disease (HSV vaccine) (n = 10,964 women; 368 pregnant)	Control vaccine (either received AS04 adjuvant alone or placebo control, or received the active comparator HAV) (n = 8763 women; 292 pregnant)	Ectopic pregnancy	Spontaneous abortion, congenital anomaly, stillbirth	NR

Note

*Denotes using subset data of adolescents and young people from studies involving participants ≥25 years of age

HAV: hepatitis A vaccine; HSV: herpes simplex virus; HPV: human papillomavirus; LBW: low birthweight; PTB: preterm birth; NR: not reported; NA: not applicable; SGA: small for gestational age

### Preconception health risk factors

#### Teenage pregnancy/young maternal age

Four reviews [36, 39–41] reported the association between young maternal age and maternal, perinatal, and child health outcomes. Among the maternal outcomes, we found a significant association between maternal anaemia and young maternal age, reporting a significant increase in the odds of maternal anaemia among adolescents aged 17 or younger (OR 1.36; 95% CI: 1.24 to 1.49; three studies) as compared to adolescents between 19 and 24 years of age [39]. A review including studies from Sub-Saharan Africa reported an increase in maternal mortality rates among adolescents ≤15 years of age compared to those who were between 20 to 24 years. But, it is essential to note that these studies were heterogeneous, with varied data collection and reporting methods [40].

Among the perinatal outcomes, we found a significant association between young maternal age (i.e., < 15 years or ≤ 15 with a low gynaecological age) and preterm birth (OR 1.68; 95% CI: 1.34 to 2.11) among those living in developing countries [39]. We also found a significant association between young maternal age (<16 years vs. 15 to 24 years) and very preterm birth (OR 1.87; 95% CI: 1.51 to 2.31) among those living in high and middle-income countries [39]. Very LBW (OR 1.39, 95% CI: 1.23 to 1.58) was also found to be associated with young maternal age (i.e., < 16 years of age) [39]. It is important to note that the OR represent a heterogenous group of women. A dose-response relationship was found between young maternal age and LBW, showing a decrease in risk of LBW with increasing age, highlighting a higher risk of LBW among babies of mothers ≤15 years of age [39, 40]. We did not find a significant impact of young maternal age on stillbirths among women from low and middle-income countries, but we found an increase in stillbirths among women living in high-income countries [39]. A significant increase in odds of clubfoot was found among babies born to mothers aged 20–24 years (OR 1.20; 95% CI: 1.05 to 1.37); while, it was observed the babies born to young mothers aged <20 years were less likely to suffer from club foot (OR 0.89; 95% CI: 0.53 to 1.52), however the impact was not significant [41]. Early neonatal (OR 29.6; 95% CI 4.4 to 199.5) and perinatal mortality (OR 1.75, 95% CI: 1.26 to 2.43) were also found to be higher among adolescents ≤15 years of age compared to those aged 20 to 24 years [40].

Only one review reported on the impact of young maternal age on child health outcomes [36]. The review suggested that children of young adolescent mothers <20 years (OR 0.88, 95% CI: 0.74 to 1.04, n = 764, I^2^ 64%) were less likely to suffer from type 1 diabetes (T1DM) compared to those born from older adolescent mothers aged 20–25 years (OR 0.95; 95% CI: 0.89 to 1.00; n = 3919, I^2^ 20%).

Lastly, it is important to note that many of these findings come from a single study. See **Table 3** for the summary of estimates.

**Table 3:** Impact of preconception health risk factors and interventions.

Outcome	Study ID		Risk factors		Interventions			
	Main review	Individual study from the included review	Maternal age	Non-communicable diseases (i.e., impact of cancer treatment)	Nutrition supplementation	Family planning	Vaccination	
					MMN supplementation vs. placebo	Youth-friendly family services	Three doses of bivalent HPV 16/18 VLP vaccine with AS04 adjuvant vs. hepatitis A vaccine (control)	Herpes simplex virus (HSV) vaccine
Perinatal outcomes								
Abortion rate	Brittain 2015					Increase from 17.2/1,000 to 23.1/1,000 (*P value* <0.0001)		
Miscarriage	Wacholder 2010						Overall, 11.5% in the HPV arm and 10.2% in the control arm (2 trials). Miscarriage rates were 14.7% in the HPV vaccine arm and 9.1% in the control arm in pregnancies that began within 3 months after the nearest vaccination.	
	Coelho 2015						One miscarriage was reported with a prevalence interval of 22.2 (number of pregnancies not reported)	
	Tavares 2013							All pregnancies within the vaccination exposure window: RR 1.7 (95% CI: 0.7–4.6). All pregnancies: RR 1.3 (95% CI: 0.8–2.1)
Perinatal death	Grønvik 2018	Ganchimeg 2013	≤15 (n = 551) vs. 20 to 24 years (n = 10242) 7.6% vs 4.5%; **OR 1.75; 95% CI: 1.26 to 2.43**; one study					
Late fetal death	Coelho 2015						One late fetal death was reported (number of pregnancies not reported)	
Neonatal death	Gibbs 2012		<16 vs. 20 to 24 years: OR: 1.09; 95% CI: 0.98 to 1.22; I^2^ 0%; 4 studies					
Early neonatal death	Grønvik 2018	Nkwabong 2009	≤15 years vs. 20 to 24 years: **OR 29.6; 95% CI: 4.4 to 199.5;** one study For girls <17 years old: OR 6.20; 95% CI: 1.01 to 38.14; one study					
Stillbirth	Gibbs 2012		No meta-analysis was conducted (reported narratively)					
	Gerstl 2018			Pooled rate from 2 matched cohort studies: (cancer treatment modality not reported) Cancer survivors: 0.01% 95% CI: 0.00 to 0.02; n = 44; I^2^ = 0% Controls: 0.01%; 95% CI: 0.006 to 0.01; n = 106; I^2^ = 0%.				
	Tavares 2013							Review reported <0.1% stillbirths in both intervention (1/10,964) and control group (1/8763)
LBW	Gibbs 2012		‘young’ age strata (10–14 years): **OR 1.82; 95% CI: 1.60 to 2.07; I**^**2**^ **0%**; 4 studies ‘middle’ age strata (13–15 years): **OR 1.56; 95% CI: 1.31 to 1.87; I**^**2**^ **80%;** 4 studies ‘older’ age strata (14–15 years): **OR 1.42; 95% CI 1.06 to 1.89;** I^2^ 96%; 4 studies					
	Gerstl 2018			Pooled rate from 2 matched cohort studies: (cancer treatment modality not reported) Cancer survivors: 10% 95% CI: 0.09 to 0.11; n = 275; I^2^ = 75% Controls: 6%; 95% CI: 0.05 to 0.07; n = 1117; I^2^ = 96%.				
	Grønvik 2018	Adam 2009	≤16 vs. 20–24 years: 7/29 (24.1%) vs 23/203 (11.3%) OR = 2.49; 95% CI: 0.96, 6.47; one study					
VLBW	Gibbs 2012		<15 vs. >15 to 25 years: **OR 1.39; 95% CI: 1.23 to 1.58; I**^**2**^ **77%;** 8 studies					
Preterm birth	Gibbs 2012		<16 years vs. ≥16 years (up to 24 years) **OR 1.87; 95% CI: 1.51 to 2.31**; I^2^ 97%; 7 studies Among young adolescents (<15 years or adolescents ≤15 with a low gynaecological age): **OR 1.68; 95% CI: 1.34 to 2.11;** I^2^ 96%; 6 studies					
	Coelho 2015						Two cases of prematurity reported (number of pregnancies not reported)	
	Grønvik 2018	Adam 2009	≤16 vs. 20–24 years: 2/29 (6.8%) vs 29/203 (14.2%) OR 0.44; 95% CI: 0.10 to 1.97; one study					
	Gerstl 2018			Rate of preterm birth **22%; 95% CI:** **0.20–0.24**; 2 retrospective reviews, I^2^ 65%				
Birth weight	Gerstl 2018-			Any treatment modality: >2500g: **10%; 95% CI:** **0.09 to 0.11; 2 cohort studies; I**^**2**^ **= 75%** 2500–3999 g: **80%; 95% CI: 0.78 to 0.81; 2 cohort studies I**^**2**^ **= 0%**				
Congenital anomaly	Liu 2016		Clubfoot: Overall: < 20 years: OR 0.9, 95% CI: 0.53 to 1.5; I^2^ 74%; 6 case-control studies **20 to 24 years: OR 1.2, 95% CI: 1.1 to 1.4; I**^**2**^ **0%; 4 studies**					
	Coelho 2015						One case of congenital anomaly was reported with a prevalence interval of 2.3 (number of pregnancies not reported)	
	Tavares 2013							Review reported <0.1% births with a congenital anomaly in both intervention (0/10,964) and control group (4/8763)
Maternal outcomes								
Maternal mortality	Grønvik 2018	Banda 2015	Pregnancy-related mortality rate in Zambia, 10–14 years: 9338/100.000 20–24 years: 557/100.000					
		De Wet 2016	Probability of dying during pregnancy, 10–14 years: 0.0001 20–24 years: 0.0039					
		Ganchimeg 2013	≤ 15 vs. 20–24 years: 73.1/10,000 births vs 19.6/10,000 births in Sub-Saharan Africa					
		Ujah 2005	≤ 15 vs. 20–24 years: 573/100,000 total deliveries vs. approx. 500/ 100,000 total deliveries in Nigeria					
		Tessema 2017	2013 data: 10–14 years: ≈120/100,000 live births 20–24 years: ≈140/100,000 live births Higher maternal mortality rates in the years 1990 and 1995					
Ectopic pregnancy	Tavares 2013							Review reported <0.1% ectopic pregnancies in both intervention (1/10,964) and control group (0/8763)
Dysfunctional uterine bleeding	Coelho 2015						One case reported (number of pregnancies not reported)	
Anaemia	Gibbs 2012		< 17 years vs. 19–24 years: **OR 1.36; 95% CI: 1.24 to 1.49; 3 studies; I**^**2**^ **45%**					
	Abe 2016				20% anaemia in the placebo group with mean haemoglobin concentrations below the normal value (12 g/dL) and significantly lower than the supplemented group (P = 0.0018) (evidence from 1 trial)			
Change in maternal body composition- change in MUAC	Gibbs 2012		From early pregnancy to 12 weeks post-partum: Adjusted change adolescents <16 years: **-0.97; 95% CI: -1.33 to -0.60,** vs. 20–25-year-olds: **-0.40; 95% CI: -0.70, -0.10** (evidence from on study)					
Child outcomes								
Type 1 diabetes	Cardwell 2010		**Overall (30 studies):** <20 years: OR 0.88; 95% CI: 0.74 to 1.04; I^2^ 64; n = 764 20–25 years: OR 0.95; 95% CI: 0.89 to 1.00; I^2^ 20; n = 3919 **Adjusted for all confounders (30 studies):** <20 years: OR 0.89; 95% CI 0.74 to 1.07; I^2^ 67%; n = 736 **20–25 years: OR 0.93; 95% CI: 0.87 to 0.99; I**^**2**^ **20%; n = 3715.** **Subgroup** Cohort studies (5 studies): **<20 years: OR 0.80; 95% CI 0.65 to 0.99; I**^**2**^ **57%; n = 269** **20–25 years: OR 0.89; 95% CI: 0.82 to 0.96; I**^**2**^ **0%; n = 1105** Case-control studies (25 studies): <20 years: OR 0.91; 95% CI 0.73 to 1.14; I^2^ 66%; n = 495 20–25 years: OR 0.97; 95% CI: 0.91 to 1.05; I^2^ 17%; n = 2814					

#### Non-communicable diseases and chronic diseases

Only one review studied the impact of noncommunicable diseases on adverse perinatal health outcomes [38]. The review examined the long-term consequences of cancer treatment on pregnancy and birth outcomes among female cancer survivors diagnosed during childhood (aged 0 to 14 years) or adolescence and young adulthood (aged 15 to 25 years). The findings from the review suggest that mothers who underwent cancer treatment had a stillbirth rate of 0.01%, which was consistent for both cancer survivors and controls. However, there was a higher incidence of LBW babies (<2500g) among cancer survivors (10%) compared to controls (6%). The specific type of cancer treatment received by these participants was not reported. Among the subset of women who received chemotherapy alone and subsequently became pregnant (n = 973), 22% experienced preterm births (<37 weeks of gestation weeks) (95% CI: 0.20 to 0.24, two retrospective reviews, n = 265, I^2^ = 65%). For all the included studies, the median age of patients at the time of cancer diagnosis and at the time of birth was 10.5 years (range: 0 to 20 years) and 28 years (range: 22 to 45 years), respectively. The review highlights limited data on the number of pregnancy terminations and miscarriages.

### Preconception health interventions

#### Vaccination

Three reviews [37, 42, 43], reported on the impact of vaccination on maternal and perinatal outcomes. Two reported on the provision of the human papillomavirus vaccine (HPV) [37, 43], and one reported on the herpes simplex virus (HSV) vaccine [42].

Wacholder et al. [43] revealed that pregnancies occurring with an interval of less than 90 days between conception and the nearest HPV vaccination showed higher miscarriage rates. In this scenario, 13.7% of pregnancies in the HPV vaccine group and 9.2% of pregnancies in the hepatitis A vaccine (control) group experienced miscarriages. This difference in miscarriage rates was most pronounced for pregnancies conceived within 90 days after vaccination. Yet, for pregnancies that began 90 days or more after vaccination, the miscarriage rates in the two vaccine groups were similar (10.7% in the HPV vaccine group and 10.5% in the hepatitis A vaccine group). Coelho et al. [37] also reported on the incidence of dysfunctional uterine bleeding, congenital anomalies, premature births, miscarriages, and late foetal death among adolescents and young adults vaccinated with the HPV vaccine.

The review also reported on the rate of miscarriages by maternal age at conception. The review showed an increase in miscarriage rates among mothers aged 21 to 23 years at the time of conception (intervention: 11.6%; control: 11.1%) compared to those ≤20 years of age (intervention: 9.4%; control: 9.8%) [43]. The review also reported stillbirths, with 0.8% in the HPV vaccine group and 0.7% in the Hepatitis A vaccine group [43].

The review on HSV vaccination reported no difference in spontaneous abortions in pregnancies that reached completion, had a known outcome, and occurred within the HSV vaccination exposure window [42]. Similarly, for all completed pregnancies, the review did not report any difference in spontaneous abortions between the HSV vaccine and control groups. The review also highlighted a very minimal risk (<0.1%) of stillbirths, ectopic pregnancies, and births with congenital anomalies in both the intervention and control groups.

#### Nutrition supplementation

One review [34] focused on nutritional interventions and their impact on maternal health outcomes. The review reported on multiple micronutrient (MMN) supplementation among breastfeeding mothers and included two trials, of which only one trial from Brazil involved adolescents. In this trial, participants in the intervention received MMN supplementation in addition to their traditional diet, while the placebo group received a typical standard of care. The nutritional supplement consisted of 162 mg of calcium (calcium phosphate dibasic), 18 mg of Iron (ferrous fumarate), 15 mg of zinc (zinc oxide), 2 mg of copper (cupric oxide) and other vitamins and minerals. The trial reported a significant reduction in maternal anaemia at 11 weeks post-partum (*P value* 0.0018). However, the review did not report on any other maternal, perinatal, and child health outcomes.

#### Family planning

One review [35] reported on family planning intervention and its impact on perinatal health outcomes. It examined the impact of youth-friendly family planning services on reproductive health. The review included 19 studies, of which only one study reported on the abortion rate by analysing repeated cross-sectional population-based surveys, while the other studies did not report on the outcomes of our interest. The study reported a significant increase in abortion rates (17.2/1,000 to 23.1/1,000) among females aged 11–19 years. The review did not report on any other maternal, perinatal, and child health outcomes.

## Discussion

This scoping review identified 10 systematic reviews that investigated preconception health risk factors and interventions among adolescents and young adults, examining their impact on maternal, perinatal, and child health outcomes from both LMICs and HICs. This scoping review identified preconception risk factors, including young maternal age and non-communicable and chronic diseases. The reviews on preconception health interventions reported on nutrition supplementation (MMN supplementation), family planning, and vaccination.

This review primarily focuses on adolescents and young adults, yet the interventions and risk factors identified align with prevailing evidence on preconception health [7, 28]. In our findings, an increased likelihood of maternal anaemia, LBW, preterm birth, stillbirths, and maternal, neonatal, and perinatal deaths were observed among adolescent mothers. Additionally, there was an elevated risk of congenital anomalies (i.e., clubfoot) in children born to mothers aged 20 to 24 years. However, a lower risk of T1DM was identified in children born to mothers aged <20 years compared to children born to mothers between 20 and 25 years of age. A recent review supported our findings [28], reporting an increased risk of preterm births associated with adolescent pregnancies. Numerous cohort studies provided evidence of the detrimental impact of young maternal age on perinatal and child health outcomes [44–51]. Fall et al. [18] in their collaborative study involving cohorts from LMICs, reported a significant increase in LBW and preterm birth among adolescents ≤19 years compared to older mothers ≥35 years. The study also reported a significant increase in stunting and wasting at the age of two years and a higher likelihood of being overweight and obese in adulthood, which our scoping review did not specifically identify. Recognising the disadvantages associated with early motherhood, many countries, particularly those classified as LMICs, have instituted legal minimum marriage ages to mitigate risks linked to young parenthood. Beyond legal considerations, additional factors contributing to these trends encompass risky behaviours and suboptimal educational attainment, independent of socioeconomic status and country.

Our review uncovered an association between cancer treatment and adverse perinatal outcomes, including preterm birth, LBW, and stillbirths. Our findings are also consistent with published literature reporting an increased risk of preterm birth and LBW [52]. The existing literature has also highlighted the risk of spontaneous abortions, foetal malposition, and congenital abnormalities among children born to cancer survivors [52].

Reviews of observational studies have consistently reported on the impact of preconception risk factors on maternal, perinatal, and child health outcomes, which can extend across generations. However, there is a notable gap in the awareness and prevention of these risk factors. It is also important to note that most of the included reviews reported on perinatal outcomes, and very few reported on maternal and child health outcomes.

Most of the included studies explored behavioural interventions related to pregnancy planning, improving knowledge, and uptake of contraceptives. However, a limited number reported on preconception health interventions specifically designed for adolescents and young adults. Our review highlighted the effectiveness of youth-friendly family planning services, including easy access to contraceptives and counselling, in significantly reducing abortion rates. Among the interventions studied, nutrition interventions such as micronutrient supplementation demonstrated a positive impact by reducing anaemia among adolescent mothers at 11 weeks postpartum. Conversely, HPV and HSV vaccination showed little to no impact on stillbirths, ectopic pregnancies, and congenital anomalies. Interestingly, one review indicated an increased risk of miscarriages among adolescents and young adults aged 21 and 23 years of years as compared to those ≤20 years of age. Our findings are consistent with previous reviews conducted by Dean et al. [6–8] and Lassi et al. [9, 11, 30] on improving preconception health among women of reproductive age. It is noteworthy that, similar to our review, there is a scarcity of systematic reviews examining the impact of preconception health intervention on adolescents and young adults. This underscores the need for more focused research and systematic reviews in this demographic to inform targeted interventions for this critical population.

The strength of the review lies in its systematic approach to searching for papers that studied the impact of early childbearing on maternal, perinatal, and child health outcomes. Rigorous screening and extraction processes involving two reviewers were implemented to minimise discrepancies. Secondly, the review included studies from all global regions to map the evidence from global perspectives. However, the review has certain limitations. Firstly, it excluded reviews published before the year 2010 and those published in languages other than the English language, potentially leading to omission of relevant studies predating 2010 or published in different languages. This review was also limited to only published studies; therefore, publication bias is a possible limitation.

The review identified insufficient evidence on the preconception health of adolescents and young adults. The existing literature primarily focuses on women of reproductive age, and the literature targeting adolescents and young adults often lacked specific outcomes of our interest. The review did not find evidence related to pre-pregnancy weight and lifestyle, mental health disorders, substance abuse, domestic and sexual abuse, chemical and environmental exposure, genetic disorders, female genital mutilation, tobacco use before pregnancy, and on prevention of non-communicable diseases and sexually transmitted diseases across extended generations. Therefore, this scoping review highlights the need for further robust studies specifically targeting the preconception health of adolescents and young adults. It emphasises the importance of conducting trials with extended follow-ups periods to comprehensively assess the impact of preconception health interventions during adolescence on subsequent pregnancies and the health of their offspring. Lastly, the findings suggest a shift in policy focus towards early interventions starting from adolescence in both men and women. This approach is seen as more comprehensive and preventive, moving beyond the conventional emphasis on women planning to conceive. By addressing health concerns and promoting health behaviours in both genders during adolescence, policies can potentially have a more significant impact on improving maternal, perinatal, and child health outcomes. This recommendation aligns with the growing recognition of the broader societal context in shaping reproductive health and underscores the importance of holistic interventions encompassing the entire reproductive lifespan.

## Conclusion

This comprehensive review consolidates findings from 10 reviews describing the impact of preconception risk factors and interventions among adolescents and young adults on maternal, perinatal and child health outcomes. It highlights the need for more rigorous studies examining various risk factors such as nutrition, STIs, abuse, tobacco and substance use on the health of mothers and their children. Notably, the review emphasises the scarcity of robust interventional studies aimed at preventing these factors. This review emphasises the necessity for future studies to address these knowledge deficiencies in the field of adolescent and young adult preconception health.

## Supporting information

S1 Checklist Preferred Reporting Items for Systematic reviews and Meta-Analyses extension for Scoping Reviews (PRISMA-ScR) checklist. (PDF)

S1 Text Search strategy. (PDF)
