# Supplementary material for: Exploring preconception health in adolescents and young adults: Identifying risk factors and interventions to prevent adverse maternal, perinatal, and child health outcomes–A scoping review
Source: PLoS One. 2024 Apr 17;19(4):e0300177. doi: 10.1371/journal.pone.0300177 (PMC11023205; doi:10.1371/journal.pone.0300177)
Supplement: S1 Text — (PDF) [file pone.0300177.s002.pdf]

S2: Search Strategy of Medline

Ovid MEDLINE(R) ALL <1946 to November 18, 2022>

1 exp Adolescent/ or exp *Adolescent Health/ or exp Young Adult/ or (preconception health or pre conception health or pre pregnancy health or prepregnancy health or before pregnancy or pre conception or preconception or prepregnancy or preconceptional or periconceptional or before conception or prior conception or young women or young woman or young men or young man or young people or youth or teen or young people or young person* or adolesc* or preadolesc* or pre-adolesc* or juvenil* or minor* or p?ediatric* or pubescen* or pre-pubescen* or prepubescen* or puberty or teen* or youth* or high-school* or highschool*).ti,ab. 3572573

2 exp Maternal Age/ or exp Overweight/ or exp Obesity/ or exp Protein-Energy Malnutrition/ or exp Malnutrition/ or exp Anemia/ or exp Vitamin D Deficiency/ or exp Thinness/ or exp Communicable Diseases/ or exp Seizures/ or exp Epilepsy/ or exp Diabetes Mellitus/ or exp Heart Diseases/ or exp Asthma/ or exp Noncommunicable Diseases/ or exp Hypertension/ or exp Hepatitis B/ or exp Hepatitis A/ or exp Hepatitis D/ or exp Hepatitis/ or exp Hepatitis E/ or exp Hepatitis C/ or exp Cardiovascular Diseases/ or exp Chronic Disease/ or exp Neoplasms/ or exp Cystic Fibrosis/ or exp Rheumatic Heart Disease/ or exp Syphilis/ or exp Thalassemia/ or exp Kidney Diseases/ or exp Arthritis, Rheumatoid/ or exp Thrombophilia/ or exp Multiple Sclerosis/ or exp Inflammatory Bowel Diseases/ or exp Noncommunicable Diseases/ or exp Acquired Immunodeficiency Syndrome/ or exp HIV/ or exp Infections/ or exp Rubella/ or exp Measles/ or exp Mumps/ or exp Typhoid Fever/ or exp Malaria/ or exp Tuberculosis/ or exp Meningitis/ or exp Syphilis/ or exp Hepatitis/ or exp Urinary Tract Infections/ or exp Toxoplasmosis/ or exp Cytomegalovirus/ or exp Sexually Transmitted Diseases/ or exp Genital Diseases/ or exp Smoking/ or exp Alcohol Drinking/ or exp Substance-Related Disorders/ or exp Illicit Drugs/ or exp Domestic Violence/ or exp coercion/ or exp intimate partner violence/ or exp anxiety/ or exp Depression/ or exp Stress Disorders, Post-Traumatic/ or exp Neurodevelopmental Disorders/ or exp Attention Deficit Disorder with Hyperactivity/ or exp Mental Disorders/ or exp Schizophrenia/ or exp Bipolar Disorder/ or exp Dental Caries/ or exp Tooth Abnormalities/ or exp Environment/ or exp Air Pollutants/ or exp Air Pollutants, Radioactive/ or exp Air Pollutants, Occupational/ or exp Particulate Matter/ or exp Tobacco Smoke Pollution/ or exp Air Pollution/ or exp SARS-CoV-2/ or exp COVID-19/ or exp Isotretinoin/ or exp Anticonvulsants/ or exp Amphetamines/ or exp Antihypertensive Agents/ or exp Hypoglycemic Agents/ or exp Analgesics/ or exp Scopolamine/ or exp Ceftriaxone/ or exp Methyldopa/ or exp Insulin, Isophane/ or exp Antiemetics/ or exp Parasympatholytics/ or exp Antidepressive Agents/ or exp "Hypnotics and Sedatives"/ or exp Tranquilizing Agents/ or exp Betamethasone/ or exp Cephalosporins/ or exp Adrenergic beta-Antagonists/ or exp Antidiuretic Agents/ or exp Vitamin A/ or exp Female Circumcision/ or exp "Wounds and Injuries"/ or (risk* or risk factor* or an?emia or IDA or iron deficiency an?emia or iron deficienc* or weight gain or weigh loss or "overweight and obesity" or adiposity or obese or overweight or malnourish* or underweight or undernourish* or thyroid disease* or connective tissue disease or cardiovascular disorder* or high blood pressure or chronic illness* or cancer* or disability* or thales?emia or renal disease* or lupus or HIV or NCDs or infectious disease* or infection* or sexually transmitted infectious disease* or sexually transmitted infection* or genital infection* or genital disorder* or venereal disease* or venereal infection* or STD or shigellosis or acute respiratory infection* or ARI or adenovirus or Hib or pertussis or paratyphoid intestinal infection* or TB or hepatitis B or hepatitis C or UTIs or urinary infection* or alcohol misuse or Illicit drug* or Caffeine or Nicotine or marijuana or vaping or alcohol or addict* or substance abuse or drug* or drug abuse or drug consumption or cannabis or heroin or cocaine or khat or hookah or shisha or inhalant or cigarette* or e-cigarette* or sexual violence or physical

violence or violence or abuse or victimi#ation or perpetration or partner violence or dating violence or stress or psychological or behavioral or Psychiatric Illness* or Psychiatric Disease* or mental health or depress* or anxi* or schizophreni* or psych* or well-being or trauma or PTSD or ADHD or oral health or periodontal or plaque or caries or decay or dental caries or dental carries or stomatognathic disease* or dental pulp disease* or toothache* or tooth demineralization or tooth injur* or toothache or oral disease* or mouth disease* or Pollution or Aeroallergen or Environment* or traffic related air pollution or Volatile organic compounds or pollen or Nitrous oxide or Nitrogen dioxide or Carbon Monoxide or Ozone or Pollen or weed or environmental toxin* or radiation exposure or environmental substance* or coronavirus or COVID-19 or Accutane or anti epileptic drug* or anti-epileptic drug* or Methamphetamines or Inhalant* or pain killer* or antibiotic* or antispasmodic or antispasmodic agent* or antidepressant* or sedative drug* or sedative medicine* or ferrous sulphate or FeSO4 or corticosteroids* or cefalotin or cefalexin or Cephalexin or statin* or Genetic or Genetic disease* or accident* or injury or unintentional injur* or sports injur* or female genital mutilation or genital mutilation).ti,ab. 21406528

3 exp Preconception Care/ or exp Reproductive Health Services/ or exp *Adolescent Health Services/ or exp Prenatal Care/ or exp Ambulatory Care Facilities/ or exp Family Planning Services/ or exp Safe Sex/ or exp Sex Education/ or exp Birth Intervals/ or exp Early Intervention, Educational/ or *Condoms/ or *Condoms, Female/ or *Contraceptive Devices, Male/ or *Contraceptives, Oral/ or *Intrauterine Devices/ or *Antiretroviral Therapy, Highly Active/ or exp Nutrition Assessment/ or exp Diet/ or exp Exercise/ or exp Immunization/ or exp Vaccination/ or exp Immunization Programs/ or exp Preventive Health Services/ or exp Measles-Mumps-Rubella Vaccine/ or exp Typhoid-Paratyphoid Vaccines/ or exp Genetic Testing/ or exp Genetic Counseling/ or *Environmental Monitoring/ or *Biological Monitoring/ or exp Disease Management/ or exp Oral Hygiene/ or exp Dental Health Services/ or exp Dental Care/ or exp Health Promotion/ or exp Telemedicine/ or exp Community Participation/ or exp Social Support/ or exp Empowerment/ or public assistance/ or food assistance/ or medical assistance/ or social security/ or exp Healthcare Financing/ or exp Obesity Management/ or exp School Health Services/ or exp Psychosocial Intervention/ or exp Motivation/ or Rehabilitation, Vocational/ or Psychiatric Rehabilitation/ or exp Rehabilitation/ or exp Employment, Supported/ or exp Smoking Prevention/ or exp Smoking Cessation/ or exp Tobacco Control/ or (pre conception care or pre pregnancy care or prepregnancy care or health education or education or counsel?ing or awareness or counsel* or couple counsel* or community mobilization or genetic counsel?ing or genetic screening or health visit or home visit or screening or perinatal care or post abortion care or family planning or contraception or chronic care or nutrition or food basket or physical activity or IFA supplement* or supplement* or folic acid or folate or iron or multivitamin* or micronutrient or prenatal vitamin* or micronutrient supplementation or Vitamin* or mineral* or energy protein or food basket or life style modification or lifestyle modification or metabolic control or cash transfer or food voucher or conditional cash transfer* or CCT or cash voucher or UCT or unconditional cash transfer* or cash transfer or smoking cessation or drug therapy or therapy or treatment or medication* or Psychological intervention* or music therapy or emotional development or vaccin* or immuniz* or vaccine* or incentive* or intervention* or Program* or health program or dental hygiene or mouth hygiene or tooth hygiene or teeth hygiene or genetic service* or genetic screen* or genetic counsel*).ti,ab. 11483023

4 2 or 3 24418921

5 exp Systematic Review/ or (meta analysis or meta-analysis or de-novo review*).ti,ab. 387413

6 1 and 4 and 5 40822

7	exp animals/ not (exp animals/ and exp humans/)	5176994
8	6 not 7 40715	
9	limit 8 to (english language and yr="2010 - Current")	**31369**
